# The acute effect in performing common range of motion tests in healthy young adults: a prospective study

**DOI:** 10.1038/s41598-020-78846-6

**Published:** 2020-12-10

**Authors:** F. Holzgreve, C. Maurer-Grubinger, J. Isaak, P. Kokott, M. Mörl-Kreitschmann, L. Polte, A. Solimann, L. Wessler, N. Filmann, A. van Mark, L. Maltry, D. A. Groneberg, D. Ohlendorf

**Affiliations:** 1 Institute for Occupational Medicine, Social Medicine and Environment Medicine, Goethe-University Frankfurt, Frankfurt Theodor-Stern-Kai 7, Haus 9b, 60590 Frankfurt am Main, Germany; 2 Institute of Sport Science, Goethe-University Frankfurt, Frankfurt am Main, Germany; 3 Institute of Biostatistics, Goethe-University Frankfurt, Frankfurt am Main, Germany

**Keywords:** Anatomy, Medical research

## Abstract

In the application of range of motion (ROM) tests there is little agreement on the number of repetitions to be measured and the number of preceding warm-up protocols. In stretch training a plateau in ROM gains can be seen after four to five repetitions. With increasing number of repetitions, the gain in ROM is reduced. This study examines the question of whether such an effect occurs in common ROM tests. Twenty-two healthy sport students (10 m/12 f.) with an average age of 25.3 ± 1.94 years (average height 174.1 ± 9.8 cm; weight 66.6 ± 11.3 kg and BMI 21.9 ± 2.0 kg/cm^2^) volunteered in this study. Each subject performed five ROM tests in a randomized order—measured either via a tape measure or a digital inclinometer: Tape measure was used to evaluate the Fingertip-to-Floor test (FtF) and the Lateral Inclination test (LI). Retroflexion of the trunk modified after Janda (RF), Thomas test (TT) and a Shoulder test modified after Janda (ST) were evaluated with a digital inclinometer. In order to show general acute effects within 20 repetitions we performed ANOVA/Friedman-test with multiple comparisons. A non-linear regression was then performed to identify a plateau formation. Significance level was set at 5%. In seven out of eight ROM tests (five tests in total with three tests measured both left and right sides) significant flexibility gains were observed (FtF: *p* < 0.001; LI-left/right: *p* < 0.001/0.001; RF: *p* = 0.009; ST-left/right: *p* < 0.001/*p* = 0.003; TT-left: *p* < 0.001). A non-linear regression with random effects was successfully applied on FtF, RF, LI-left/right, ST-left and TT-left and thus, indicate a gradual decline in the amount of gained ROM. An acute effect was observed in most ROM tests, which is characterized by a gradual decline of ROM gain. For those tests, we can state that the acute effect described in the stretching literature also applies to the performance of typical ROM tests. Since a non-linear behavior was shown, it is the decision of the practitioner to weigh up between measurement accuracy and expenditure. Researchers and practitioners should consider this when applying ROM assessments to healthy young adults.

## Introduction

In the application of range of motion (ROM) tests there is little agreement on the number of repetitions to be measured and the number of preceding warm-up protocols. In general, ROM tests are tools to measure joint mobility on the basis of routine (scientific) procedures^1–4^. The aim of any ROM test is to determine the maximum joint mobility. Hence, in every execution of the test, a stretching stimulus is applied on the connected muscle–tendon units. It has been shown, that in stretch training, such an acute effect occurs after only a few repetitions and manifests itself among other things in increased mobility, stretch tolerance and reduced passive torque^4–9^. It can be expected that this acute effect also occurs during the ROM test. Therefore, differences in the measurement protocol with regard to the measured repetitions and previous warm-up exercises, could lead to different results. However, the current evidence does not allow a determination of precise measurement protocols.

With a reproducible test procedure, the ROM value is a parameter to show changes in flexibility in an intervention. Depending on the joints to be measured, measuring tapes, goniometers or inclinometers are usually applied^10–12^. In general, ROM assessments depict either the active ROM or the passive ROM. To perform active ROM, the person to be tested moves the assessed joint without assistance using the agonistic musculature. In passive ROM, the therapist, the investigator, or another external force, moves the body part of the person to be tested through the ROM^1^. However, changes in passive torque, stretch tolerance and isometric muscle force influence the ROM value.

In static and dynamic stretching, an acute effect occurs within the first five stretches for ROM, stretch tolerance, passive torque and energy, which then return to baseline after 1 h^5,8,9^. The acute effect of stretching on ROM shows the greatest improvement in the first repetitions, whereas the ROM gain is reduced with increasing repetitions^4,7^. Consequently, four to five repetitions are recommended for the practical implementation within a stretch training session since subsequent gains are only minimal^6,7^. Accordingly, a logarithmic behaviour can be attributed to the acute effect of stretching on ROM. This is valid for stretching training, but how does it behave when the ROM is to be determined in an assessment setting?

The current literature provides no answers on whether such an effect occurs in the performance of common ROM tests. Scientific practice uses mixed approaches for the problem of acute effects. For example, in interventions and normative data surveys, one to three repetitions were carried out with no to two trials' warm-ups, mostly without any information on rest times^13–17^ or with no information about the number of repetitions^18,19^. In most reliability studies, either one repetition was primarily carried out^10,20,21^ or a warm-up on a bicycle ergometer employed, followed by one to two trials of warm-up prior to the actual measurement being conducted^22,23^. In a reliability study investigating trunk mobility, each subject was instructed to perform five repetitions of each tested motion in advance of the measurement being taken^24^.

In conclusion, there is no homogeneity regarding warm-up, the number of repetitions and averaging in the context of interventions, normative data surveys and reliability studies. Particularly, in light of a possible acute effect of stretching on ROM, a standardized procedure is necessary, since in common stretching the effect is very large, especially in the first five repetitions^4,6,7^. Due to the inconsistent implementation, in both the practical application and in reliability studies, the presence of an acute effect harms the comparability of ROM test results. Also, if it can be assured, that subjects are in a warmed up state, possible stiffening factors (e.g. sports prior to measurement) can be controlled for.

Thus, the aim of this study was to investigate the repetition-dependent acute effect of stretching on the ROM in the application of ROM tests and whether an equal behaviour can be derived. One further goal was to test whether there is a plateau formation after several repetitions. Therefore, multi-joint movements were chosen because they are restricted by the muscle–tendon unit. Single-joint movement are on the other hand limited by bones (e. g. elbow extension), mass (e. g. knee flexion) or ligaments (e. g. knee extension)^25^. Five frequently used and evaluated multi-joint ROM tests were selected, which mainly evaluate the mobility of the trunk: Fingertip-to-Floor test^1,26^, Lateral Inclination test (left and right side)^1,27^, Retroflexion of the trunk after Janda^1^, Shoulder test after Janda (left and right side)^1,28^ and the modified Thomas test (left and right side)^1,10,29^. On this basis, recommendations can be derived for the practical application of quality criteria.

## Material and methods

### Subjects

Twenty-two healthy sports students (10 m/12 f.; 25.3 ± 1.94 years; 174.1 ± 9.8 cm; 66.6 ± 11.3 kg; 21.9 ± 2.0 kg/m^2^) volunteered in this prospective study. Exclusion criteria were relevant operations or surgical stiffening of the musculoskeletal system, a relevant artificial joint replacement, severe diseases such as ankylosing spondylitis, chronic destructive joint diseases, multiple sclerosis, myodystrophic or neurodegenerative diseases, congenital malpositions of the musculoskeletal system or an acute herniated disc. In addition, the intake of muscle relaxants or other drugs that influence the elasticity of the musculature and pregnancy were considered as contra indicators. Two sports students with experience in exercise physiology carried out the measurements; both raters were instructed on the methods and practised until the execution was satisfactory.

All participants provided written informed consent to take part in the study in advance. This study was approved by the ethics research committee of the Goethe-University (2018–46) in Frankfurt am Main, Germany and was conducted in accordance with the 1964 Helsinki Declaration and its later amendments.

### Measurement systems and ROM tests

**Figure 1:**
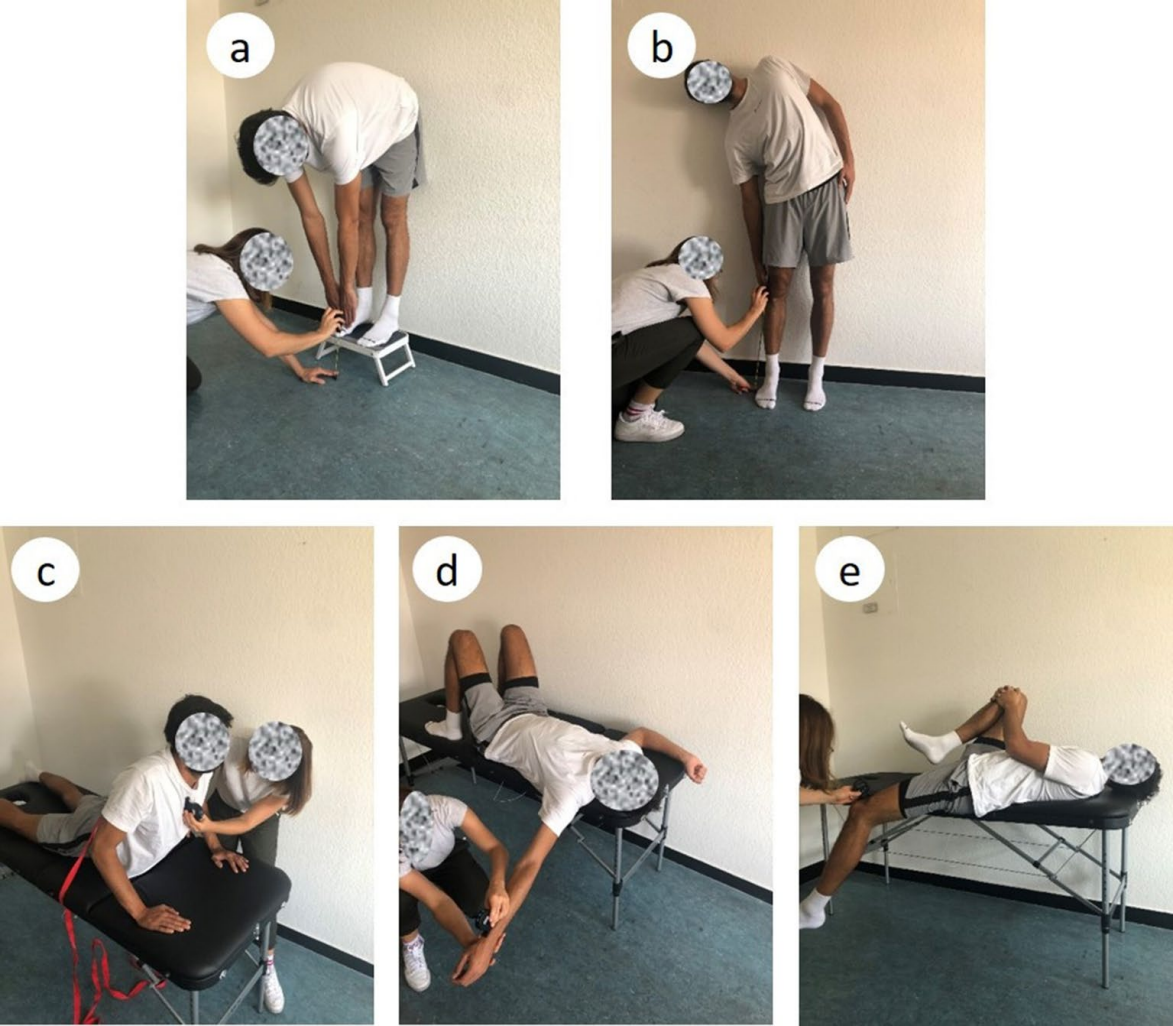
The ROM tests examined in this study. (**a**) Fingertip-to-Floor test; (**b**) Lateral Inclination test; (**c**) Retroflexion of the trunk after Janda in a modified version; (**d**) Shoulder test modified after Janda; (**e**) Modified Thomas test.

The ROM measurements used in this study, which are described below, were evaluated with either a tape measure (Fingertip-to-Floor and Lateral Inclination test)^26,30–33^ or a digital inclinometer (Retroflexion, Shoulder and Thomas test)^10,27,29,34–41^ (Fig. 1). The digital inclinometer (Model: Acumar Digital Inclinometer Model ACU002 / Lafayette Instrument Company / Lafayette / USA) has a measurement accuracy comparable to a goniometer^35^. As the digital inclinometer shows only integers, the absolute measurement error was set to 0.3°. A detailed description of the measurement tools and the ROM tests can be found in Holzgreve et. al.^15^.

#### Fingertip-to-floor test (FtF)

The FtF test is used to assess the active ROM of the back, both hips, the ischiocrural musculature and the neuromeningeal structures^1^ (Fig. 1a). A tape measure is used to assess the distance between the most distal point of the fingers and the floor. Accordingly, a smaller measure corresponds to a greater flexion performance. The reliability of this test lies between r = 0.76 and r = 0.99^20,30–32^ and shows a good sensitivity for changes^26^.

#### Lateral inclination (LI)

This test is performed in a standardized stand position by flexing the upper body in the frontal plane (Fig. 1.b). Sagittal fluctuations in the Lateral Inclination are eliminated by leaning the back against a wall. The trunk lateral flexion active ROM is evaluated with a tape measure measuring the distance between the fingertip and the floor^1,27^ and has an intrarater reliability of 0.95^42^.

#### Retroflexion of the trunk after Janda in a modified version (RF)

In order to evaluate the extension of the lumbar and thoracic spine, the modified retroflexion test according to Janda (RF) was performed^28^ (Fig. 1c). The participants lay on a treatment couch with a tensioning strap located at the level of the posterior superior iliac spina, counteracting pelvic rotation in the sagittal plane. With the hands placed next to the shoulders the participants push and extend their elbows as far as possible. The position of the thoracic spine in the sagittal plane is determined by placing the digital inclinometer on the sternum. This test evaluates the spinal active ROM^1^.

#### Shoulder test modified after Janda (ST)

This shoulder mobility test is a modification of the examination after Janda^28^ (Fig. 1d). The ST is a passive ROM test^1^ which evaluates the mobility of the shoulder joint, especially of the pectoralis major muscle. For this purpose, the subjects where positioned on the bench in a supine position. The arm is extended in 90° abduction. In contrast to Janda, the elbow is stretched. The digital inclinometer is then placed proximal to the processus styloideus radii on the radius.

#### Modified Thomas test (TT)

The pelvic inclination must be controlled to obtain valid results^29^ (Fig. 1e). In order to standardize the pelvic inclination, the digital inclinometer is placed downwards of the anterior superior iliac spine. In this position, the alignment of the pelvis is set to 0°. The extension inclination is then measured by placing the inclinometer on the thigh above the patella. The modified Thomas test is a widely used passive ROM test to assess the presence of hip flexion contracture and to measure hip extensibility^1,29^. For both the digital inclinometer and goniometer, high interrater reliabilities have been reported (r = 0.91–0.93; ICC = 0.89–0.92)^10^. The intrarater parallel-forms reliability is also very high with correlations of r = 0.91–0.93; ICC = 0.89–0.92^10^.

### Procedure

There was no standardized warm-up, because every repetition represents a specific warm-up. All subjects performed all testing in one day. There was no familiarization prior to the testing. Each subject performed 20 repetitions in each ROM test listed above. The test order was randomized. Each test repetition had to be held on the active or passive maximum ROM (according to the test) for about three seconds. The investigator counted down from three, when the maximum ROM position was reached. At zero, the examiner recorded the ROM according to each test protocol. There was a break of 3 s between each repetition in each testing session. All measurements were carried out by two raters.

### Statistical analysis

Statistical analysis was performed using BiAS version 11.08 (Epsilon-Verlag, Darmstadt, Germany) and R (R Core Team 2019)^43^ and figures were produced using the package ggplot2^44^. Since no such study is known to date from which expected results can be derived and, in particular, no information on the dispersion of the data is available, no formal power analysis is carried out. The study described here is therefore to be regarded as a pilot study. To determine if an acute effect occurs at the individual level, a regression was performed on every individual’s performance. Subsequently, the number of subjects with a slope sign corresponding to an ROM gain were counted for each ROM test. According to normal distribution of the data, either univariate ANOVA with repeated measurements^45^ or the Friedman-test^46^ with multiple comparisons were conducted. These tests were performed in order to identify an overall effect and changes within 20 repetitions. Bonferroni correction was used to counteract the problem of multiple comparisons. The mean of the standard error of measurement of each repetition served as a parameter for the measurement accuracy. If the sign test was positive, non-linear regression was applied. Random effects took repeated measurements into account, thus describing the behavior of a potential acute effect of ROM: a*exp(-b*x) + c.

With "a" being the amplitude at the very beginning, "b" the coefficient describing the change from trial to trial and "c" the asymptotic measure. In case, the model was not adequate (i.e. parameter estimations were non-significant), linear regression with random effects was performed to quantify the trend: a·x + b.

Significance was set at 5%.

### Ethics approval

All participants provided written informed consent to take part in the study in advance. This study was approved by the ethics research committee of the Goethe-University (2018–46) in Frankfurt am Main, Germany.

## Results

Except of TT-right (*p* = 0.93), all ROM tests revealed significant flexibility gains within 20 repetitions, indicating changes of ROM due to repetitive stretching (FtF: *p* < 0.001; LI-left/right: *p* < 0.001/*p* < 0.001; RF: *p* = 0.009; ST-left/right: *p* < 0.001/*p* = 0.003; TT-left: *p* < 0.001) (Table 1). Furthermore, multiple comparisons revealed the first significant ROM gain in FtF, LI-left/right, RF and ST-left after five to eight repetitions, whereas in ST-right the first significant gain occurred after 19 repetitions. However, TT-left and TT-right showed no significant changes in multiple comparisons. All repetitions were held for 3 s.

**Table 1:** P-values of Friedman test and ANOVA for each ROM test.

	Fingertip-to- floor test	Lateral Inclination		Retro-flexion	Shoulder test		Mod. Thomas test	
		Left	Right		Left	Right	Left	Right
Friedman/ANOVA	*p* < 0.001^1^	*p* < 0.001^1^	*p* < 0.001^1^	*p* 0.01^2^	*p* < 0.001^1^	*p* < 0.01^1^	*p* < 0.001^1^	*p* > 0.05^1^
individuals w/ROM gain	22/22	18/22	20/22	16/22	20/22	19/22	16/22	13/22
Mean of standard error of measurement	1.555	0.664	0.665	0.818	1.65	1.989	1.185	1.564
50% of movement	5	5	5	2	6	-	6	-
75% of movement	10	9	9	3	11	-	11	-

Relative amount of individuals with an ROM gain, mean of the standard measurement error and number of repetitions needed to achieve 50% and 75% of the total ROM increase for each ROM test, respectively.

ANOVA = 1 ; Friedman test = 2.

In the individual analysis, only FtF showed a uniform effect, indicating a ROM gain for every volunteer. Furthermore, in LI-left/right and ST-left/right more than 80% of the subjects experienced a ROM gain (Table 1). In contrast, in RF and TT-left/right less than 75% of the subjects recorded a ROM gain (Table 1).

Furthermore, mean values of the standard error of measurement in contralateral sides differ in ST (1.65/1.989) and TT (1.185/1.564), whereas LI shows almost identical mean values of the standard error of measurement (0.664/0.665) (Table 1).

**Figure 2:**
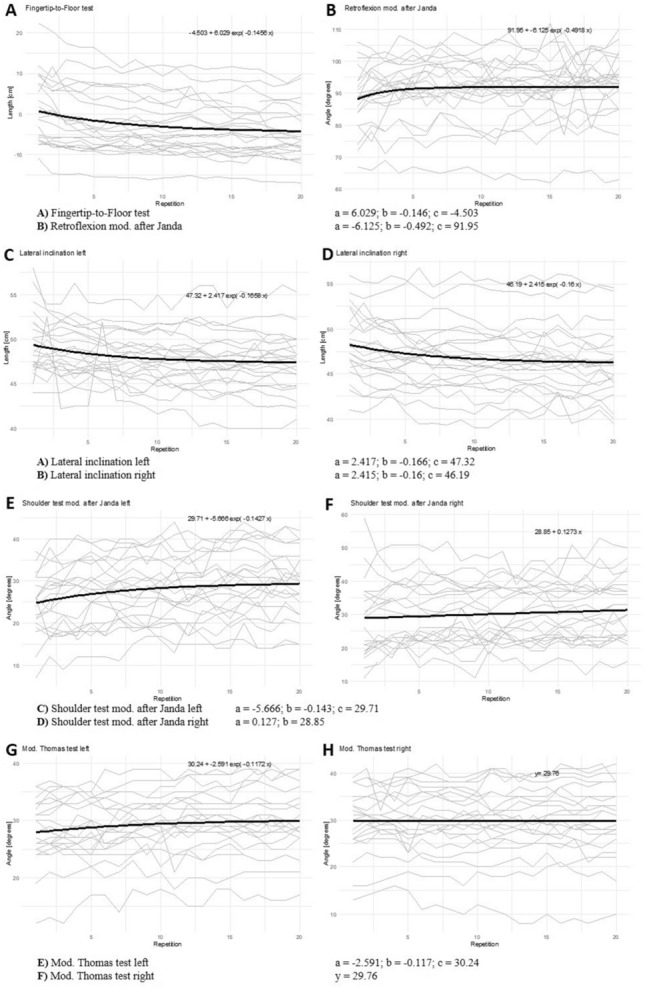
Non-linear regression for FtF, RF, LI, ST left and TT left. Linear regression for ST right and TT right. Parameters of the function are shown below.

The non-linear regression for FtF, RF, LI-left/right, ST-left and TT-left showed a repetition dependent gradual decline in the ROM gained (Fig. 2). Hence, a plateau formation could be derived from the non-linear regression. As the non-linear model was not adequate for ST-right and TT-right a linear regression with random effects was performed to quantify the trend (Fig. 2). Whereas in ST-right a slope of at least 0.127 of ‘a’ could be derived, there was no significant difference between ‘a’ and zero for TT-right.

The parameter estimates LI-left and -right were significantly different for the parameter ‘a’ (*p* < 0.0001). Having successfully applied a non-linear regression on six out of the eight ROM tests, it is possible to postulate predictions on the number of repetitions needed to achieve a certain amount of maximal ROM gain (Table 2).

**Table 2:** Relative ROM gain dependent on the number of repetitions for each ROM test.

% total ROM increase	Fingertip-to-floor test	Lateral inclination		Retroflexion	Shoulder test		Mod. Thomas test	
		Left	Right		Left	Right	Left	Right
25	3	2	2	1	3	-	3	-
50	5	5	5	2	6	-	6	-
60	7	6	6	2	8	-	7	-
70	9	8	8	2	10	-	9	-
80	12	10	11	3	13	-	12	-
90	17	14	15	4	18	-	17	-
95	22	19	19	5	24	-	23	-
99	32	28	29	8	36	-	34	-

## Discussion

Except of TT-right all ROM tests demonstrated a repetition-dependent change in ROM. In addition, six out of the eight ROM tests (FtF, RF, LI-left/right, ST-left and TT-left) showed a gradual decline in the amount of ROM gained, indicating a plateau formation (Fig. 2). The results confirm the hypothesis that ROM tests provide a stretch stimulus analogous to stretching exercises. They are consistent with repetition-dependent acute effects of stretch training^4,6,7,47,48^ and demonstrate that this effect is also present in typical ROM tests. Based on the conducted regression, a plateau could be derived for each ROM test, which depicts an area where ROM gain is very small and negligible with each further repetition^4^. The increase in mobility lays between 1 and 6 degrees and 2 and 6 cm, respectively (Fig. 2). Thus, it can be stated that a different number of repetitions performed in mobility assessment settings lead to different angle values for the ROM (Fig. 2).

For the practical application of these six ROM tests, the regression provided information on the number of repetitions above which the test offers reliable values for the evaluated ROM (Table 2). This is especially the case when different or unknown warm-up states are employed; the execution of several repetitions according to the regression can allow for a better standardization. The number of repetitions required for a certain increase in ROM is very similar amongst ROM tests which supports the stability of the applied methods.

With respect to the practical application, we recommend to assess in the area of the plateau of each test. Yet a concrete naming of a plateau should be avoided since, even in higher stretch repetitions, small gains were recognizable. However, increasing the ROM from 50% of maximal to 60% of maximal required only one or two repetitions (e. g. FtF/LI in Table 2). When approaching the plateau, more repetitions were needed to gain the same amount of ROM, for instance, to increase from 80 to 90% up to five additional repetitions were necessary (Table 2). The respective plateau formation can be derived descriptively from Fig. 2. Only the RF differed significantly from the other results. The goodness of fit of RF (adjusted R squared: 0.606; root mean square error: 0.746) was clearly worse than those in the other tests; this may be due to the fact that performing the RF requires maximum elbow extension, therefore, fatigue effects cannot be excluded when performing 20 maximum repetitions.

Although the type of stretching used in all ROM tests was static, the duration differed greatly from the duration used in static stretching. While in common ROM tests the subjects keep their positions for seconds, recommendations for static stretching range from five seconds to 15 min or even more^49–54^. Boyce and Brosky^6^ showed a plateau occurrence for 15 s static stretching at the fifth through to the tenth stretch repetition.

The reason why an overall effect on the individual level could not be shown is probably because the effects are very small in comparison to the measurement accuracy. In total, the range of variation of the measurements is large, especially for the digital inclinometer which only uses integers. Here, one sees differences favoring the tape measure over the digital inclinometer regarding individual performances.

In the present investigation, only the regression curve of RF (Fig. 2) appeared to match the proposed plateau formation timing. In the FtF, the plateau formation (80% of the total ROM increase) of the LI-left and right, ST-left and TT-left occurred between the 10th and 13th stretch repetition (Table 2). The largely uniformly delayed plateau formation compared to Boyce and Brosky's^6^ may be explained by a significantly shorter stimulus exposure time. There appears to be dose-dependent differences, although the underlying effect is the same. The fact that in RF the plateau was already reached by the fifth stretch repetition, may be a result of fatigue effects occurring in performing this ROM test. Furthermore, RF has less muscle inhibition of the movement compared to the other ROM tests investigated. The spine mobility is mainly limited by ligaments, especially the ligamentum longitudinale anterius concerning hyperextension^55^. Due to their collagen structure, ligaments have only limited stiffness^56^. A limitation of motion due to ligament inhibition may explain premature plateau formation in RF.

Tests such as FtF, TT and LI displayed high intrarater reliabilities (FtF: r = 0.76–0.99^26,29–32^; TT: r = 0.89–0.92^10,29^; LI: r = 0.95^42^), which were mostly determined in the first trial over two days^10,20,21^. Due to mostly good reliabilities, it can be assumed that the warm-up state of the subjects was standardized. Nevertheless, it is unclear as to how sensitive the measurement of the first repetition is for changes, e.g. in the context of an intervention. Does the first repetition represent the actual mobility? In addition, the question arises as to what extent sample measurements, different warm-up states, special warm-ups or faulty measurements with subsequent second repetitions falsify the results. Since the influence of additional repetitions on the ROM in the context of ROM tests has scarcely been investigated so far, reference must be made at this point to further, future investigations. Angle values obtained from protocols that differ in the application of the number of repetitions, preliminary warm-up routines or averaging must not be compared. It should be noted that the functions (Fig. 2) provide the basis for a mobility measurement on the plateau. This could serve as a basis for the comparability of ROM tests.

No exponential regression could be performed in ST-right and TT-right, whereas it could be applied for the contralateral side. Considering the mean values of the standard error of measurement for each ROM test, Table 1 shows discrepancies among contralateral sides in ST and TT. The mean standard error of measurement of the right side was considerably greater; this may explain the inconsistent data for the right side in ST and TT. In both tests, which measure the mobility of extremities, the right side showed little or no difference. The discrepancy may be due to the one-sided distribution of handedness and footedness. Biomechanical differences have been shown between left- and right-handed baseball pitchers and also for the elbow flexion, horizontal glenohumeral abduction and wrist coronal plane motion^57^. Furthermore, discrepancies in the test protocol existed in TT and ST compared to FtF, LI and RF. In TT there was no offset visible between the measurements during the test execution. The subjects remained in a permanent strain with sub-maximal intensity. The duration of stimulus exposure was, therefore, significantly longer than in the active ROM tests, in which the stimulus was maintained for about 3 s. In addition, in ST the arm to be measured was moved through and repositioned after each measurement repetition. However, these discrepancies do not explain different contralateral results, but may lead to different intertest outcomes There are further differences between the ROM tests in the different strain methods implicitly used in the test execution that may lead to different outcomes. If the ROM tests are categorized according to the properties and effects of stretching, then differences between the tests become apparent (Table 3).

**Table 3:** Stretching properties (stretching type, torque type, source of torque, intensity and physiological cause for increased ROM) for each ROM test.

	Fingertip-to-floor	Lateral inclination	Retroflexion	Shoulder test	Thomas test
Stretching type	Static	Static	Static	Static	Static
Torque type	Active	Active	Active	Passive	Passive
Source of torque	Antagonist and gravity	Antagonist and gravity	Antagonist and elbow extension	Gravity	Gravity
Intensity	Maximal	Maximal	Maximal	Sub-maximal	Sub-maximal
Physiological cause for increased ROM	Muscle/tendon stiffness ↓ stretch tolerance ↑	Muscle/tendon stiffness ↓ stretch tolerance ↑	Muscle/tendon stiffness ↓ stretch tolerance ↑	Muscle/tendon stiffness ↓	Muscle/tendon stiffness ↓

Differences in either active or passive stretching are particularly remarkable^1^. While FtF, LI and RF tested at maximum torque, ST and TT tested at sub-maximal torque. The source of torque in ST and TT derived solely from gravity and depended on the weight, length and weight distribution of the lever-arm. As the effects between maximal and sub-maximal stretching on the ROM differed^47^, this, may explain the discrepancies.

As our subjects were young healthy adults, further studies are needed to exploit whether this effect does also occur in impaired populations or in the elderly.

## Conclusion

An acute effect was observed in most ROM tests, which is characterized by a gradual decline in the amount of ROM gain. Thus, the same number of repetitions is required for the increase in ROM of 0–50% as for the increase of 50–75% of the total ROM increase. The behaviour of this acute effect could be determined using a non-linear regression for most of the ROM tests. For these tests, we can state that the acute effect described in the stretching literature of ROM also applies to the performance of typical ROM tests. Researchers and practitioners should consider this when applying ROM assessments to young healthy adults.

## Data Availability

There are no further data or materials than shown in this manuscript.

## Abbreviations

ROM: Range of motion
FtF: Fingertip-to-floor test
LI: Lateral inclination test
RF: Retroflexion of the trunk modified after Janda
TT: Modified Thomas test
ST: Shoulder test modified after Janda
